# Understanding the meanings of male partner support in the adherence to therapy among HIV-positive women: a gender analysis

**DOI:** 10.1080/16549716.2022.2051223

**Published:** 2022-04-13

**Authors:** Isotta Triulzi, Claire Somerville, Salimu Sangwani, Ilaria Palla, Stefano Orlando, Hawa Sangare Mamary, Fausto Ciccacci, Maria Cristina Marazzi, Giuseppe Turchetti

**Affiliations:** a Institute of Management, Scuola Superiore Sant’Anna, Pisa, Italy; b Gender Center, Graduate Institute of International and Development Studies, Geneva, Switzerland; c Dream Programme, Community of Sant’Egidio, Blantyre, Malawi; d Department of Biomedicine, University of Tor Vergata, Rome, Italy; e UniCamillus, Saint Camillus International University of Health Sciences, Rome, Italy; f Department of Human Science, Lumsa University, Rome, Italy

**Keywords:** gender, HIV/AIDS, sexual and reproductive health, partner’s support, male involvement

## Abstract

**Background:**

Previous literature reports that low male partner support is a barrier to women’s adherence and retention in HIV care programs.

**Objective:**

This qualitative study explored the relationships between partners to understand what is meant by male partner support in adherence of HIV-positive women in four healthcare facilities in Southern Malawi.

**Methods:**

We conducted 8 semi-structured focus group discussions (FGDs) with 73 participants (40 men and 33 women) and 10 in-depth interviews (IDIs) between August 2018 to December 2019. Participants were HIV-positive patients, healthcare workers (HCWs), expert patients (EPs), and couples attending the clinic. All data were digitally recorded, transcribed verbatim, and analysed using a gender-responsive grounded theory approach.

**Results:**

This study confirms previous literature, which suggests male partner support is expressed by providing access to transport to the clinic and accompaniment to appointments. However, we found that men can also control access to resources and decision-making. Support is more complex than previous literature reported and, in some cases, gender norms significantly limit women’s capacity to engage in care independently of male support since women need male partner permission to access the resources to attend clinics.

**Conclusions:**

This paper suggests that restrictive male-partner gender norms limit women’s power to engage in care. Most importantly, the gender analysis reveals that what previous literature describes as male partner support can sometimes hide male partner control in permitting access to resources to attend health facilities. For this reason, policies enhancing male support should consider the gender power relationship between partners to avoid reinforcing gender inequality.

## Background

Malawi has reported the highest HIV prevalence for men and women 25+ years adults in the world [1]. AIDS is a major cause of death for adults and children under five in Malawi [2] with annual HIV-related deaths of 13,000 people among a population of 18 million people [3]. Keeping patients engaged in long-term therapy is a critical element of HIV care to achieve the suppression of Viral Load, prevent treatment failure and avoid the development of drugs-resistance. In Malawi, 70% of the 29,313 women who started Prevention of Mother to Child Transmission (PMTCT) with antiretroviral therapy (ART) under Option B+ were still in care three years later [4].

The barriers to adherence to care in sub-Saharan Africa are clearly described in several studies where male partner support plays an important role in retaining women in therapy [5,6], especially in antenatal care [7–9]. Evidence suggests that interventions enhancing male involvement would improve uptake of maternal ART, infant prophylaxis uptake [10,11], HIV testing [12], reduction of infant HIV infection [13], as well as changes in couple relationships such as increased couple communication, relationship quality and equitable decision-making [14,15]. Some interventions in Tanzania and Zimbabwe foster love and intimacy in couple relationships leading to an enhancement in men’s practical support for maternal and child health and domestic duties [16]. Male partner support is also reported to alleviate the social and economic difficulties associated with the disease and facilitate adherence [15,17].

The WHO and other public health organizations promote male partner involvement as a strategy to improve women’s adherence and uptake of PMTCT [18]. However, little is known about how women themselves view and experience male partner support or the underlying gendered assumptions about how this support operates. We aim to understand what is meant by male partner support from the perspectives of women and men partners, expert patients (EPs), and healthcare workers (HCWs) in the adherence of women in HIV care in the context of household gender roles and norms.

## Methods

### Study setting and population

This qualitative study was embedded in a larger study (WeMen study) investigating the factors affecting male partner support among HIV-positive women in Malawi including interventions to enhance male partner support in care [19,20].

Malawi is one of the poorest countries in southern Africa, with a low population density and more than four-fifths of its people living in rural areas. Nearly 80% of the population is employed in the agriculture sector [21]. The largest ethnic group are Chewa (34.4%) followed by Lomwe (18.9%), Yao (13.3%), and Ngoni (10.4%); 75% of the population is Christian, and 25% of it practice the Muslim religion and other beliefs [22]. Systems of kinship vary and both matrilineal and patrilineal systems are found in different areas of the country [23,24]. For example, in the Southern region where our study took place, most districts are described as matrilineal [25]. Nevertheless, households are mainly headed by men, and village political structures are generally led by men with a few exceptions [26]. Our study is located in an area where there is a recognised gendered division of labour whereby men are mainly responsible for household finances, whilst women are in charge of the domestic sphere [27,28]. Recent data suggest that there is a high prevalence of gender-based violence [29]

We conducted semi-structured focus group discussions (FGDs) and in-depth interviews (IDIs) with women and men couples, HIV-positive patients attending the clinic, expert patients, and healthcare workers. HIV-positive expert patients are trained to provide peer counselling, psychosocial support, and assistance in accessing and adhering to HIV treatment. They aim to create a supportive bridge between community and healthcare facilities. The composition of FGDs was varied to triangulate design and maximise perspectives. All the patients were on ART. Patients or couples, who attended the clinic the same day, were invited to participate in this study and were recruited by clinic directors. The clinic director invited all the health personnel working in the healthcare center to participate in FGDs and IDIs including expert patients and healthcare workers. The study was conducted at four healthcare facilities located in Southern Malawi: Blantyre, an urban area, and rural areas of Namandanje, Balaka, and Kapire. These health facilities are part of the DREAM programme, a public health initiative focused on HIV/AIDS, TB and NCDs [30].

### Data collection

Data and information were collected between August 2018 and December 2019. The first authors, CS, and IT designed the protocol. A local researcher (SS) moderated discussions and provided contextual socio-cultural facilitation and IT facilitated the discussion. Each FGDs lasted between 60 and 90 minutes, and IDIs between 30 to 60 minutes. The number of participants in the FGDs ranged from six to eleven. The protocol developed for the study explored experiences of HIV as a couple and its impact and effect on the household. We investigated the following areas:

1. Meaning of support for women and their partners, HCWs, and EPs
2. Household roles concerning health, access to resources, decision making and the division of labour, including childcare.

The study integrated a gender perspective in the design with the addition of questions aimed at eliciting responses to gendered roles, norms, and relations at the houselhold level. Some questions were adapted from IMAGES survey methodology [31] to the Malawian context.

The protocol was validated during the first two FGDs. We conducted interviews in the local language, Chichewa, or in English or both languages depending on the participants’ preferences. The interviews and focus groups were recorded and transcribed verbatim and translated into English by a local researcher. The interviews were anonymous: we assigned a number to each participant, and we requested verbal informed consent to proceed with the IDIs or FGDs before starting the recording. Data collection continued until the research team agreed we had achieved saturation.

### Data analysis

We adopted an inductive approach derived from grounded theory [32]. We reviewed the transcripts to become familiar with the text and discussed and described emerging themes. Drawing out these open-coded themes we developed an initial extensive codebook and grouped gender codes to assist with sorting [33]. The first round of coding was open-ended in a constant comparative process [34] after which the codebook was then piloted on two transcripts, reviewed, and revised. Data were uploaded to RQDA package (R) a qualitative package that supports coding and data management. We reviewed the transcripts line-by-line and systematically assigned codes. After finalizing the coding, we reviewed all the previous analyses and sorted data to the point of saturation under second order themes. The themes and their supporting data were iteratively discussed and interpreted by IT and CS. IT, CS, IP were involved in addressing the organizational aspects of the study and the process of analysis. IT and CS analysed and interpreted the data, held regular meetings during the process of analysis to discuss and distill interpretations.

## Results

We recruited 73 participants for FGDs and IDIs. Tables 1 and 2

**Table 1.** Description of FGDs participants

	Participants	Number of FGDs	Number of participants (F;M)	Age range	Location
FGDs	Couples where both partners are HIV-positive	1	6 (3;3)	27–50	Blantyre
	Couples with only one HIV-positive partner	1	6 (3;3)	21–41	Blantyre
	Women	1	6 (6;0)	38–54	Blantyre
	Men	3	23 (0;23)	29–62	Namandanje, Balaka
	Healthcare workers*	1	11 (3;8)	26–45	Blantyre
	Expert Patients	1	11 (10;1)	28–45	Blantyre
	Total		63		

*Two medical Doctor; six nurses; one counselor; one clinic director; one medical assistance

**Table 2.** Description of interviews participants

		Number of participants	Age range	Location
IDIs	Expert clients	5	38–51	Kapire
	Women	2	41	Blantyre
	Men	3	48–65	Blantyre
	Total	10		

describes their characteristics.

### Meaning of male support for women

We identified two main activities associated with male partner support for women: accompanying women to clinics and providing access to transport. Significantly, we found two sets of divergences: first, a divergence between the type of support women desired, and what they received; second, a divergence of views between how men reported supporting, and the support women said to receive. HCWs and EPs reported that they view male partners accompanying women to appointments as an act of support. We explored the divergences through the two male partner activities identified as support.

#### Accompanying women

Women expressed a strong desire for male partner support as an expression of love and, in the main, welcomed the opportunity for partners to attend the clinics with them. Attending the clinic with their female partner is considered by women as a responsibility of husbands and a demonstration of their love for partners.

The husband should show us love, like the way my husband was doing, although he wasn’t coming, he used to encourage me and remind me to come here or when he comes to taking drugs, he reminds me. Love is important because you feel encouraged.(Woman, 39, FGD women)

For example, this woman expressed such sentiment:

It’s the responsibility of a husband to take us to the hospital, that make us feel like our husbands is loving us. Even if we might not have anything to eat but if husbands are loving us everything seems okay.

(Woman, 42, FGD women)

However, we found that only a minority of men attended the clinics with their partners: just 3 out of 9 male FGD participants in Balaka. Barriers to male attendance were described by women and HCWs and included: stigma and negative partner reaction after disclosure, lack of privacy in the clinic, precarious conditions and male income generation activities, and absence of secure space for a bike. Policy incentives have included the use of fines in some villages to incentivize men to attend the clinic, although it has not been an effective approach since some reported they preferred to pay the fee rather than accompanying the woman. Some male partners did recall their positive role in attending clinics and efforts to support wives in different contexts.

How should we support women? Supporting women is not an issue. If the woman is sick, as men we should come in to help them by taking the child to the hospital.(Man, 35, FGD couples)

We would do everything together or even coming here we would come together to receive the drugs.(Man, 45, FGD couples)

Whilst others offered a variety of reasons and explanations as to why they did not typically join their female partners in the clinic, for example:

Our education level and the way our tradition taught us it is something unusual for a man to escort his wife to the antenatal service apart from the fact that men are usually busy. Traditionally, that is impossible for husbands to escort their wives.(Man, 43, FGD male)

Clinic staff encouraged the idea that male partner support was provided by the accompaniment to the clinic. This is reinforced at the government clinic where a policy to prioritize women who are accompanied by partners is used to promote the practice. We encountered two policy initiatives that aimed to encourage male partners to accompany women to the clinic as an act of support to increase adherence to care.

When the woman is pregnant, we tell the man to be escorting the wife to the hospital and that they should both go for HIV testing. We insist to them that PMCT is not only for women, but both the husband and wife should have a shared responsibility.

(Man Expert patients, 51, IDI)

Resistance to male attendance at clinics also extended to the health care needs of children and in the following quote, the woman emphasized gender norms around care roles affect support.

I think it’s our tradition here even if the woman is working and finding a lot of money people take it as an abomination for a husband to take a child to the hospital. They think that the man is just doing that to impress the woman because she has a lot of money.(Women, 34, FGD couples)

From my personal experience I think the first reason is most men don’t know how to carry a child. When such a thing happen people may stop them and comment on how they have carried their child and in so doing men think that they have lost their value. The second reason is that most men don’t know what to do when a child is crying as the woman is at home maybe it could be that the child has urinated and men don’t know the change the urine on the child.

(Man, 51, FGD couple)

#### Access to healthcare facilities

Distance from healthcare facilities can be a significant barrier to female retention in clinics in the rural areas where this study was conducted. Partner support was expressed by providing transport or financial resources to travel. Allocating money to transport was not necessarily easy in this context when faced with competing financial needs and a recurrent theme in our study revolved around gendered decisions in the allocation of limited financial resources.

Women should be helped in terms of transport money because if your husband leaves you 500 kwacha, as a mother, I would prefer my kids to eat before I consider coming to the facility(Women, 38, FG women)

Most of the people here in the rural area mostly use bicycles or walk. So they just support their wives with some money to buy some snacks and so on.

(Man, Expert Patient, 43, IDI)

Male partners expressed their support in economic terms, as one husband said:

I leave most of the money at home, with her and if she tells me that she is sick I tell her to go to the hospital using that money. If she falls sick while am at home, I escort her to the hospital

(Man, 46, FGD couple)

Support varied with the degree of acceptance of female partner HIV status and household roles in couples. Generally, greater HIV-status acceptance by men and more equitable division of household labour were associated with increased support.

When my wife was found positive whilst I was found negative, I became very supportive to her. I was like her guardian reminding her to take her medication and follow instructions. And when I was found positive too, I told her that look we go into this together, let us be strong.(Man, 59, FGD male)

When we wake up before anything we pray, then we start sweeping the compound with my husband after that I divide mopping the house and cleaning plates between our children. In general, we help each other even when am not around and the children are not around he cooks nsima for us. Interviewer: I want to understand because it is a rare thing, how did this happen? Interviewee: it depends with the way you love each other, but for everything to work out I just trust prayer. On your own, you can’t manage but when you are praying God takes all the things you are praying for. So if you keep on praying asking God to change your husband eventually God answers your prayers. Interviewer: So it means if your child is sick your husband can take him /her to the hospital? Interviewee: yes he does.

(Woman, 36, IDI)

Lower levels of acceptance of female HIV status were linked to lack of transport support. Extreme and sometimes violent responses to the disclosure of female partner HIV status can result in the collapse of a household with the suggestion that women may be chased back to their home village and left without any economic support for health.

Another cause is the way these women are being treated by the husband when they went back home when they have been tested HIV positive. After having been tested and they are HIV-positive, they have to start drugs, they have to bring the new to the clinic. Some are being chased away from the house, the partner said that he is ok, she has to leave the house and go back to home village. Maybe they live in the town and they should go to the their village with is difficult for them to reach a clinic to take the drugs, so they just stop taking the drugs and attending the clinic. (Women, Nurse, 43, FGD Healthcare worker)

The woman is regarded as the one who has contracted the virus and so she is regarded as wicked. Since she is the one found positive first, the man thinks she was promiscuous. So, chasing her is like removing the dirt. (Man, 59, FGD male patients)

### Gender barriers in household

We identified two main gendered barriers to women’s adherence and retention in HIV care: access to resources and decision-making.

#### Access to resources

Women’s autonomy and status in the household are determined by several factors including access to education, employment, and resources. Among our participants, we found there were many more opportunities for these among men than for women, as reported in this quote:

Most women undermine themselves here in Malawi. Even when it comes to business, we have the mentality that men can do better than us in terms of the amount earned per month. As a result, women are the ones who remain at home because she finds less money

(Women, 33, FGD couples)

Men have priority in economic decision-making and expenditure as they are typically the primary or only source of income. Very few women generate cash income, although they may have access to some cash sums given to them by partners for small purchases. Participants in this study reported that hiding salary and failing to provide enough money to support the family was the primary cause of disagreements within couples. Men expressed fear that female partners might cheat them of money and spend it inappropriately. Conversely, women emphasized that men spend income in extramarital relationships or drinking alcohol, as illustrated in this quote.

It becomes a problem for a woman to find something to give the sick child whilst the man has gone to the bar to drink beer. That’s like the problem we are facing here in Malawi in many locations. In addition to that only women take part in childcare and it’s as if the woman is staying alone whilst she is married.

(Women, 41, IDI)

Whilst women have a role in food and clothing expenditures, male partners decide on major purchases. Husbands may give wives some share of their income, but typically we found that women can spend it only after obtaining permission.

She is afraid that if she goes alone, she will be shouted at because she went without his permission. The problem goes on until it worsens and if the neighbours ask her, she says I can’t go without my husband’s permission or I can’t take money in the house without his permission(Man, Expert Patient, 27, FGD)

Ignorance I mean, some of people in communities they are illiterate, if they went to school may be majority, they just attended class one to three, so it is very hard for them to understand. … Yes, so that’s among the reasons. Sometimes could be culturalism, whenever there is gathering in the community you will find that men are just sending their women to attend even if the chief called for a meeting. I feel like it’s a perception, even going to hospital with a child, always is a woman who goes.(Men, Expert Patient, 33, IDI)

#### Participation in decision-making

Women and men reported that men were the primary decision-makers in the family, derived in part from their role in income generation as breadwinner.

If you are positive, but you have a job and you have money, things are fine. But if you don’t have a job and it turns out that you are positive, things may change. You lose your respect in the family because you are taken as inferior in the family. People are waiting for you to provide for them because a man is a provider, if you don’t provide you cease being a breadwinner.

(Man, 25, FGD male)

The quote, among many others we collected, illustrated how social pressure and gender norms construct masculinities that can challenge the provision of support to female partners. Households are typically male-headed and decision-making power accompanies this role. In the case of disagreement within the couple, one woman reported that her ‘husband stops buying food and things become difficult’ (Female, 23, IDI). Male partner gender roles and decision-making power can impact access to health.

I am the one who makes the decision. I make sure that kids should go to school, I feed them and clothe them as well. When any of us is sick I have to make a decision to go to the hospital. She tells me how she is feeling in her body and when am told I have to make a decision for her to go to the hospital before the condition continues.

(Man, 49, IDI)

In most cases, women had little control over their treatment decisions and access to the clinic because this was a male partner’s decision. With few exceptions, women in this study sought permission or at least informed their partners before deciding to access health facilities. For this reason, the meaning of male support of women’s access to health facilities can be as much about permission as it is about support.

Sometimes you may find that the child is sick, but the woman is failing to take her to the hospital because the husband is not around. She is afraid that if she goes alone she will be shouted because she went without his permission. So, when the husband comes back he might find the child playing and if the wife tries to tell him that the child is sick he thinks that his wife wants to steal money from him. The problem goes on until it worsens and if the neighbours ask her she says: “I can’t go without my husband’s permission or I can’t take money in the house without his permission”. (Men, 32, FGD couples)

A woman reported that when her child was sick, she failed to go to the hospital because her husband was not around to inform. This highlights the gendered nature of decision-making in couples as shown in the following quotes.

Some women do not understand how families go like and how to handle men. I think a good wife has to inform her family that she is not feeling okay and she should get a permission from the husband. She is free to go to the hospital even without the permission, but she has to use all means necessary to inform the husband.(Man, 51, FGD couples)

In families people have different characters some are humble, some are not and they don’t understand how families should go like. Some women are rude if the child is sick she will just take him to the hospital without even informing the husband and when you ask the reason why they answer rudely. Sometimes it happens that you haven’t gone for work if she falls sick you can just inform your boss that you will there late you want to take your wife to the hospital. In families there are different protocols in some the wife can phone the husband if he is at work to inform him that she is going to the hospital and the husbands agrees with the idea and tells her that if the sickness worsens tell me to come and take care of you. There are different kind of families some understand each other, some don’t.

(Man, 36, FGD couples)

Male decision making extended also to access to contraception and family planning services as illustrated by this HIV-positive woman:

In families people have challenges, more especially women. They fail to have transport money and some basic needs. Some families hardly greet one another. Men don’t care bearing a lot of children even though they know the status of the wife or even if the woman doesn’t want to have a child (Woman, 41, IDI).

Further still, gender-based violence in the home was frequently reported by women during informal discussions with the author, further undermining any limited decision-making power.

### Discussion

This study provides insight into male partner support in the adherence of women in HIV care. Previous literature reported that men have a significant role in supporting women remain in treatment [7,15,35,36]. Our findings confirm the literature reporting that male support is promoted by HCWs and understood as providing transport access and accompanying women to the clinic.

Previous studies in Kenya, South Africa, and Malawi suggest a range of different types of interventions to encourage male partner support can increase women’s attendance at clinics and adherence to therapies [15,37,38]. Our finding confirms that male support entails accompaniment and enabling access to the health facility as previously reported by various studies in Sub-Saharan Africa (Kenya, Uganda, Malawi) [36–41] [42, 43–55].

Our gender analysis challenges some of the assumptions in the literature around the meanings behind acts of male partner support. We highlight that underlying gender norms significantly limit women’s ability to engage independently of male support, and that women’s access to health is complicated by gender relations where women need to ask for support in terms of transport resources to attend the clinic. This departs from the literature in that what has been understood as acts of support given by male partners is also about women seeking permission from male partners. It is less that women need male support, but perhaps more that without it, they do not have the decision-making power or resources to attend clinics. Male support masks the need for male partner permission reflecting unequal and gendered relations of power in couples. The act of support, from a male partner, may be an act of permission from a female partner perspective. Male partner support acts as a gatekeeper to women’s health access, and for some women, it is experienced as a form of male control. For this reason, interventions to increase male partner support of women may produce unintended and harmful consequences. Interventions to encourage male partner support should consider whether they reinforce male power over women’s health as part of a gender analysis [46,47].

However, gender relations are not fixed and are constantly changing. Conroy A et al. showed that young people are challenging some gender norms in Malawi, and creating space for transformative gender agendas that challenge gender norms [48]. Furthermore, Schatz E (2005) has challenged the prevailing discourses on female vulnerability in Malawi showing that married women have some agency in preserving their health [49], and gender analyses should be cognisant of changes in local contexts.

Engaging men as HCWs and EPs may play a crucial role if gender-specific training is provided to raise awareness on the importance of considering these underlying gender roles and norms. A recent study conducted in South Africa suggested that meaningfully involving more men in care, such as HCWs, has the potential to improve men’s clinical outcomes, discuss and shift masculine norms related to care [50].

Evidence shows that programs discussing and questioning gender norms and the social construction of masculinity (gender-transformative) positively influence relationship quality and promote gender-equitable relationships [51,52], that effect health outcomes [53,54]. Previous interventions rarely considered power dynamic as decision-making and how men and women get in relationships, rather they adopted a reductionist approach without addressing the underlying gender norms [56].

Strength and limitation

We note several limitations of this study. Participants were selected from willing men, women, and couples contacted at the healthcare facilities by the clinical coordinators. There were challenges in recruiting couples with only one HIV-positive partner since some partners were not available to take part in the FGDs. Finally, since recruitment occurred at the facilities there may be some context-specific bias in recruitment. To mitigate against clinic-context bias and create a safe research environment, the facilitator and moderator were not involved in clinical practices.

The strengths of the study stem in part from its interdisciplinary team and multiple data sources. Researchers are from different disciplines such as medical anthropology and gender (CS), public health and qualitatitave method (SS), natural science and qualitative method (IT). Local language knowledge was important to the study. The data collection was carried out in four different periods of time within this study enabling an iterative research process of analysis, data collection and interpretation. This gave us the time to examine the data collected, read and reread them, revise the codes and concepts accordingly, and reach a depth of insight.

### Conclusion

This gender analysis suggests that male partner support of HIV-positive female partners can also be understood in some cases as male partner permission to access resources to attend clinic facilities. Interventions to encourage male-partner support of HIV-positive women may have the unintended effect of reinforcing harmful gender norms in which male partners are gatekeepers to health facility access. Enhancing male support without considering the gender power relationship between partners may reinforce gender inequality and generate unintended consequences. Gender analysis can bring into light the gender norms that affect women’s retention to care.

## Author contributions

IT & CS conceived the study. IT, CS, IP, SS, SO and TG were involved in the acquisition, analysis or interpretation of data. IT drafted the manuscript. CS, IP,SS and SO revised it critically. All authors read and approved the version to be published.

## Paper context

Low male partner support is a barrier to women’s adherence and retention in HIV care programs. Using a gender-relevant grounded theory approach, we found that support is more complex than previous studies reported, and in some cases is an issue of male partner permission to access the resources to attend clinics in Southern Malawi. Enhancing male support without recognizing the gender power relationship could reinforce gender inequality between partners.

## References

[1] AIDS Data Repository. Malawi 2020 Spectrum file. version 9 - Spectrum-File. 2019 [cited 2020 Nov 24]. Available from: https://adr.unaids.org/spectrum-file/malawi-2020-spectrum-file-version-9

[2] United Nation AIDS. Malawi | UNAIDS. 2019 [cited 2020 Apr 6]. Available from: https://www.unaids.org/en/regionscountries/countries/malawi

[3] The World Bank. Malawi data. 2018 [cited 2020 Nov 26]. Available from: https://data.worldbank.org/country/MW

[4] Haas AD, Tenthani L, Msukwa MT, et al. Retention in care during the first 3 years of antiretroviral therapy for women in Malawi’s option B+ programme: an observational cohort study. Lancet HIV. 2016 Apr 1;3:e175–9. 27036993 10.1016/S2352-3018(16)00008-4 PMC4904064

[5] Omonaiye O, Kusljic S, Nicholson P, et al. Medication adherence in pregnant women with human immunodeficiency virus receiving antiretroviral therapy in sub-Saharan Africa: a systematic review. BMC Public Health. 2018;18. 10.1186/s12889-018-5651-y PMC6020364 29945601

[6] Gourlay A, Birdthistle I, Mburu G, et al. Barriers and facilitating factors to the uptake of antiretroviral drugs for prevention of mother-to-child transmission of HIV in sub-Saharan Africa: a systematic review. J Int AIDS Soc. 2013 Jul;16:18588. 23870277 10.7448/IAS.16.1.18588 PMC3717402

[7] Gugsa S, Potter K, Tweya H, et al. Exploring factors associated with ART adherence and retention in care under option B+ strategy in Malawi: a qualitative study. PLoS One. 2017;12:e0179838. 28636669 10.1371/journal.pone.0179838 PMC5479573

[8] Phiri N, Tal K, Somerville C, et al. “i do all i can but i still fail them”: health system barriers to providing option B+ to pregnant and lactating women in Malawi. PLoS One. 2019;14:1–16. 10.1371/journal.pone.0222138 PMC6742345 31513684

[9] Kim MH, Zhou A, Mazenga A, et al. Why did I stop? Barriers and facilitators to uptake and adherence to ART in option B+ HIV care in Lilongwe, Malawi. PLoS One. 2016;11:e0149527. 26901563 10.1371/journal.pone.0149527 PMC4762691

[10] Takah NF, Kennedy ITR, Johnman C. Impact of approaches in improving male partner involvement in the prevention of mother-to-child transmission (PMTCT) of HIV on the uptake of PMTCT services in sub-Saharan Africa: a protocol of a systematic review and meta-analysis. BMJ Open. 2016;6:1–7. 10.1136/bmjopen-2016-012224 PMC4947788 27371555

[11] Takah NF, Atem JA, Aminde LN, et al. Male partner involvement in increasing the uptake of infant antiretroviral prophylaxis/treatment in sub Saharan Africa: a systematic review and meta-analysis. BMC Public Health. 2018;18:1–12. 10.1186/s12889-018-5171-9 PMC5812221 29439695

[12] Baiden F, Remes P, Baiden R, et al. Voluntary counseling and HIV testing for pregnant women in the Kassena-Nankana district of northern Ghana: is couple counseling the way forward? AIDS Care - Psychol Socio-Medical Asp AIDS/HIV. 2005 Jul;17 648–657. 10.1080/09540120412331319688 16036251

[13] Aluisio A, Richardson BA, Bosire R, et al. Male antenatal attendance and HIV testing are associated with decreased infant HIV infection and increased HIV-free survival. J Acquir Immune Defic Syndr. 2011;56:76–82. 21084999 10.1097/QAI.0b013e3181fdb4c4 PMC3005193

[14] Davis J, Luchters S, Holmes W. Men and maternal and newborn health: benefits, harms, challenges and potential strategies for engaging men. Melbourne/Australia: Compass: Women’s and Children’s Health Knowledge Hub.; 2012. Women’s and Children’s Health Knowldge Hub

[15] Conroy A, Leddy A, Johnson M, et al. ‘I told her this is your life’: relationship dynamics, partner support and adherence to antiretroviral therapy among South African couples. Cult Heal Sex. 2017 Nov 2;19:1239–1253. 10.1080/13691058.2017.1309460 PMC5626574 28398134

[16] Comrie-Thomson L, Mavhu W, Makungu C, et al. Male involvement interventions and improved couples’ emotional relationships in Tanzania and Zimbabwe: ‘when we are walking together, I feel happy. Cult Health Sex. 2020 Jun 2;22:722–739. 31429674 10.1080/13691058.2019.1630564

[17] Watt MH, Maman S, Earp JA, et al. “It’s all the time in my mind”: facilitators of adherence to antiretroviral therapy in a Tanzanian setting. Soc Sci Med. 2009 May;68:1793–1800. 19328609 10.1016/j.socscimed.2009.02.037 PMC3530387

[18] Implementation of Option B+ for Prevention of Mother-To-Child Transmission of HIV: the Malawi experience. World Health Organization. https://apps.who.int/iris/handle/10665

[19] Triulzi I, Keiser O, Somerville C, et al. Social determinants of male partner attendance in women’s prevention-of mother-to-child transmission program in Malawi. BMC Public Health. 2020 Dec 30;20:1821. 33256655 10.1186/s12889-020-09800-4 PMC7708238

[20] Orlando S, Palla I, Ciccacci F, et al. Improving treatment adherence and retention of HIV-positive women through behavioral change interventions aimed at their male partners: protocol for a prospective, controlled before-and-after study. JMIR Res Protoc. 2021;10:e19384. 33492232 10.2196/19384 PMC7870353

[21] World Bank. Malawi overview: development news, research, data. [cited 2022 Jan 31]. Available from: https://www.worldbank.org/en/country/malawi/overview#1

[22] WorldAtlas. Religious Beliefs In Malawi. [cited 2022 Feb 27]. Available from: https://www.worldatlas.com/articles/religious-beliefs-in-malawi.html

[23] Chimbiri AM. The condom is an “intruder” in marriage: evidence from rural Malawi. Soc Sci Med. 2007;64:1102–1115. 17240504 10.1016/j.socscimed.2006.10.012

[24] Peters PE. Against the odds. matriliny, land and gender in the Shire Highlands of Malawi. Crit Anthropol. 1997;36:3–6.

[25] Mtika MM, Doctor HV, Matriliny, patriliny, and wealth flow veriations in rural Malawi. African Sociol Rev. 2002;6:71–97.

[26] Conroy AA. “It means there is doubt in the house”: perceptions and experiences of HIV testing in rural Malawi. Cult Heal Sex. 2014;16. 10.1080/13691058.2014.883645 PMC3991518 24580127

[27] Tawfik L, Watkins SC. Sex in Geneva, sex in Lilongwe, and sex in Balaka. Soc Sci Med. 2007;64:1090–1101. 17123678 10.1016/j.socscimed.2006.10.002

[28] CultureGrams World Edition. Republic of Malawi. 2018.

[29] NSO-Malawi, DSHProgram. Malawi demographic and health survey 2015-16. Zomba/Malawi: National Statistical Office; 2017.

[30] Ciccacci F, Tolno VT, Doro Altan AM, et al. Noncommunicable diseases burden and risk factors in a cohort of hiv+ elderly patients in Malawi. AIDS Res Hum Retroviruses. 2019;35:1106–1111. 31468993 10.1089/AID.2019.0125

[31] Barker G, Greene M, Nascimento M, Segundo M, Ricardo C, TaylorA, Aguayo F,et al. Men Who Care : A Multi-Country Qualitative Study of Men in Non-Traditional Caregiving Roles. Washingoton D.C; International Center for Research on Women(ICRW) and Rio de Janeiro: Instituto Promundo. March 2012.

[32] Lingard L, Albert M, Levinson W. Qualitative research: grounded theory, mixed methods, and action research. Bmj. 2008;337:459–461. 10.1136/bmj.39602.690162.47 18687728

[33] Glaser B. Grounded theory and gender relevance. Health Care Women Int. 2002;23:786–793. 12487694 10.1080/07399330290112317

[34] Strauss A, & Corbin J. (1994).Grounded theory methodology : An overview.In N. K. Denzin & Y. S. Lincoln (Eds.),Handbook of qualitative research. 273–285. Sage Publications, Inc

[35] Flax VL, Yourkavitch J, Okello ES, et al. ``If my husband leaves me, I will go home and suffer, so better cling to him and hide this thing{“} : the influence of gender on option B plus prevention of mother-to-child transmission participation in Malawi and Uganda. PLoS One. 2017;12:e0178298. 28594842 10.1371/journal.pone.0178298 PMC5464556

[36] Mulewa P, Satumba E, Mubisi C, et al. “I was not told that i still have the virus”: perceptions of utilization of option B+ services at a health center in Malawi. J Int Assoc Provid AIDS Care. 2019;18. 10.1177/2325958219870873 PMC6900569 31478427

[37] Maman S, Moodley D, Groves AK, Defining male support during and after pregnancy from the perspective of HIV-positive and HIV-negative women in Durban, South Africa. J Midwifery Women’s Heal. 2011;56:325–331. 10.1111/j.1542-2011.2011.00029.x PMC3137135 21733102

[38] Nyondo-Mipando AL, Chimwaza AF, Muula AS. “He does not have to wait under a tree”: perceptions of men, women and health care workers on male partner involvement in prevention of mother to child transmission of human immunodeficiency virus services in Malawi. BMC Health Serv Res. 2018;18:187. 29554917 10.1186/s12913-018-2999-8 PMC5859523

[39] Mabachi NM, Brown M, Sandbulte Matthew WC, et al. Using a social support framework to understand how HIV positive Kenyan men engage in PMTCT/EID care: qualitative insights from male partners. AIDS Behav. 2020;24:18–28. 30877581 10.1007/s10461-019-02451-6 PMC6745277

[40] Rogers AJ, Weke E, Kwena Z, et al. Implementation of repeat HIV testing during pregnancy in Kenya: a qualitative study. BMC Pregnancy Childbirth. 2016;16. 10.1186/s12884-016-0936-6. PMC4940827 27401819

[41] Muwanguzi PA, Nassuna LK, Voss JG, et al. Towards a definition of male partner involvement in the prevention of mother-to-child transmission of HIV in Uganda: a pragmatic grounded theory approach. BMC Health Serv Res. 2019;19:1–11. 31399088 10.1186/s12913-019-4401-x PMC6688339

[42] Mkandawire E, Hendriks SL. A qualitative analysis of men’s involvement in maternal and child health as a policy intervention in rural central Malawi. BMC Pregnancy Childbirth. 2018;18:1–12. 29351778 10.1186/s12884-018-1669-5 PMC5775573

[43] Aborigo RA, Reidpath DD, Oduro AR, et al. Male involvement in maternal health: perspectives of opinion leaders. BMC Pregnancy Childbirth. 2018 2;18:3. 29291711 10.1186/s12884-017-1641-9 PMC5749010

[44] Kululanga LI, Sundby J, Malata A, et al. Male involvement in maternity health care in Malawi. Afr J Reprod Health. 2012;16:145–157. 22783678

[45] Hampanda KM, Mweemba O, Ahmed Y, et al. Support or control? Qualitative interviews with Zambian women on male partner involvement in HIV care during and after pregnancy. PLoS One. 2020;15:e0238097. 32853263 10.1371/journal.pone.0238097 PMC7451516

[46] Connell R. Masculinities. Cambridge: University of California Press; 1995.

[47] Courtenay WH. Constructions of masculinity and their influence on men’s well-being: a theory of gender and health. Soc Sci Med. 2000;50:1385–1401. 10741575 10.1016/s0277-9536(99)00390-1

[48] Conroy A, Ruark A, Tan J. Re-conceptualising gender and power relations for sexual and reproductive health: contrasting narratives of tradition, unity, and rights. Cult Health Sex. 2020 Apr 20;22:48–64. 10.1080/13691058.2019.1666428 PMC7170748 31633456

[49] Schatz E, “Take your mat and go!”: rural Malawian women’s strategies in the HIV/AIDS era. Cult Heal Sex. 2005;7:479–492. 10.1080/13691050500151255 16864217

[50] Gittings L, Grimwood A, We need other men to stand up and start the journey’ engaging men as HIV community health workers - a gender transformative approach? Cult Heal Sex. 2020;23:192–206. 10.1080/13691058.2019.1700306 PMC7483154 32133938

[51] Barker G, Ricardo C, Nascimento M, et al. Questioning gender norms with men to improve health outcomes: evidence of impact. Glob Public Health. 2010;5:539–553. 19513910 10.1080/17441690902942464

[52] Stern E, Pascoe L, Shand T, et al. Lessons learned from engaging men in sexual and reproductive health as clients, partners and advocates of change in the Hoima district of Uganda. Cult Health Sex. 2015;17:190–205. 10.1080/13691058.2015.1027878 PMC4706030 25953243

[53] Tokhi M, Comrie-Thomson L, Davis J, et al. Involving men to improve maternal and newborn health: a systematic review of the effectiveness of interventions. PLoS One. 2018;13:1–16. 10.1371/journal.pone.0191620 PMC5784936 29370258

[54] Ruane-mcateer E, Amin A, Hanratty J, et al. Interventions addressing men, masculinities and gender equality in sexual and reproductive health and rights: an evidence and gap map and systematic review of reviews. BMJ Glob Heal. 2019;4:1634. 10.1136/bmjgh-2019-001634 PMC6747894 31565410

[55] Montgomery E, Van Der Straten A, Torjesen K. Male involvement in women and children’s HIV prevention: challenges in definition and interpretation. J Acquir Immune Defic Syndr. 2011;57:114–116. 10.1097/QAI.0b013e31821d33d6 21860358

[56] Comrie-Thomson L, Tokhi M, Ampt F, et al. Challenging gender inequity through male involvement in maternal and newborn health: critical assessment of an emerging evidence base. Cult Health Sex. 2015;17:177–189. 10.1080/13691058.2015.1053412 PMC4706017 26159766

